# Improved natural melanin production by *Aspergillus nidulans* after optimization of factors involved in the pigment biosynthesis pathway

**DOI:** 10.1186/s12934-022-02002-0

**Published:** 2022-12-30

**Authors:** William Bartolomeu Medeiros, Kelly Johana Dussán Medina, Sandra Regina Pombeiro Sponchiado

**Affiliations:** 1 Department of Biochemistry and Organic Chemistry, Institute of Chemistry, Sao Paulo State University (UNESP), Araraquara, SP 14800-060 Brazil; 2 Division of Microbial Resources - Research Center for Agriculture, Biology, and Chemical, University of Campinas – UNICAMP, Campinas, SP, 13083-970, Brazil; 3 Department of Engineering, Physics, and Mathematics, Institute of Chemistry, Sao Paulo State University (UNESP), Araraquara, SP 14800-060 Brazil

**Keywords:** Melanin, Response surface methodology, *Aspergillus nidulans*, l-DOPA, Fungal pigment

## Abstract

**Background:**

Melanin is a natural pigment that can be applied in different fields such as medicine, environment, pharmaceutical, and nanotechnology. Studies carried out previously showed that the melanin produced by the *mel1* mutant from *Aspergillus nidulans* exhibits antioxidant, anti-inflammatory, and antimicrobial activities, without any cytotoxic or mutagenic effect. These results taken together suggest the potential application of melanin from *A. nidulans* in the pharmaceutical industry. In this context, this study aimed to evaluate the effect of factors L-tyrosine, glucose, glutamic acid, l-DOPA, and copper on melanin production by the *mel1* mutant and to establish the optimal concentration of these factors to maximize melanin production.

**Results:**

The results showed that l-DOPA, glucose, and copper sulfate significantly affected melanin production, where l-DOPA was the only factor that exerted a positive effect on melanin yield. Besides, the tyrosinase activity was higher in the presence of l-DOPA, considered a substrate required for enzyme activation, this would explain the increased production of melanin in this condition. After establishing the optimal concentrations of the analyzed factors, the melanin synthesis was increased by 640% compared to the previous studies.

**Conclusions:**

This study contributed to elucidating the mechanisms involved in melanin synthesis in *A. nidulans* as well as to determining the optimal composition of the culture medium for greater melanin production that will make it possible to scale the process for a future biotechnological application.

**Supplementary Information:**

The online version contains supplementary material available at 10.1186/s12934-022-02002-0.

## Background

Melanin is a natural dark pigment found in animals, plants, and microorganisms, often playing a vital role in defense and protection mechanisms that improve the survival and competitiveness of organisms in unfavorable environmental conditions. Apart from protection against UV radiation, this pigment exhibits a myriad of biological functions, such as antioxidant, anti-inflammatory, immunomodulatory, antimicrobial, antitumoral, and anti-aging [1–3].

Owing to its multifunctionality, high biocompatibility, and biodegradability, melanin has been recognized as a promising biomaterial for numerous biotechnological applications in the biomedical, pharmaceutical, technological, and environmental fields [4]. For example, thin melanin films can be applied as a semiconductor material on different devices [5], nanoparticles of melanin can be used as nanocarrier drug release [6], and melanin can be incorporated in cosmetic formulations for the protection against oxidative skin damages [7–10] and this pigment can also act as an efficient biosorbent for metal removal/recovery from wastewater [11].

Among all organisms, fungi play a valuable and eco-friendly source of natural pigments, including melanin, because they can produce high yields of pigment easily at a low cost, making the bioprocess economically viable on the industrial scale [1, 12–15]. Fungi synthesize different types of melanins by oxidative polymerization of phenolic or indole compounds, such as glutaminyl-3,4-dihydroxybenzene (GDHB), 3,4-dihydroxyphenylalanine (DOPA), 1,8-dihydroxy naphthalene (DHN) or catechol.

In a previous study, we characterize the pigment produced by *Aspergillus nidulans* as 3,4-dihydroxyphenylalanine (DOPA)-melanin according to its physicochemical properties and tests with melanin biosynthesis inhibitors [16]. We also showed that this pigment exhibits antioxidant activity, neutralizing free radicals generated by biological oxidants as hypochlorous acid, and anti-inflammatory activity, acting as an inhibitor of NO and TNF-α production in macrophages stimulated by bacterial lipopolysaccharide [9, 10]. In addition, we demonstrated that melanin from *A. nidulans* did not exert a cytotoxic effect on the McCoy cells even in the presence of a metabolic activation system, and also has no mutagenic activity for the *Salmonella Typhimurium* strains [17]. These results indicate that the melanin produced by the *mel1* mutant may be a promising material for the development of new therapeutic agents.

Given the wide range of melanin applications, the optimization of cultivation conditions is necessary to improve the performance of the process, increasing yields and reducing costs for large-scale industrial production. For this, it is important to identify the nutritional and environmental factors that have the greatest influence on melanin production by *mel1* mutant. As proposed by Bell and Wheeler [18], the biosynthesis pathway for fungal DOPA-melanin is initiated by the conversion of l-tyrosine or l-DOPA to dopaquinone catalyzed by laccase and tyrosinase enzymes, which require the binding of copper ions in the catalytic center for enzymatic activity [1, 19, 20]. Thus, the exogenous supply of substrates and enzymatic cofactors involved in the melanin biosynthesis pathway may stimulate its production. Sun et al. [21] reported that there is a positive correlation between tyrosine concentration and melanin production in *Auricularia auricular.* Eisenman et al. [19] also observed that melanization of *Cryptococcus neoformans* occurs rapidly after the addition of l-DOPA in the culture and increasing the concentration of l-DOPA promotes greater melanin formation. Galhaup and Haltrich [22] observed that melanin content in the mycelia of *Trametes pubescens* was higher when it was grown in the presence of copper.

In a previous study, some nutritional and environmental parameters were already evaluated and the following optimized condition for melanin production by the *mel1* mutant was established: 10^6^ conidia. mL^−1^ and 1% corn steep liquor in the 1st step; 10% pre-inoculum, 0.2% corn steep liquor, and 0.2% Tween in the 2nd step; pH adjusted to 6.8 and a constant agitation speed of 225 rpm at 29 ºC [23].

In this way, this study aimed to evaluate the effect of L-tyrosine, glucose, glutamic acid, l-DOPA, and copper ions on melanin production and to determine their concentrations optimal for maximizing melanin production by the *mel1* mutant using response surface methodology (RSM). After optimization of the medium composition, the production of melanin by *mel1* mutant increased by 640% compared to the previous condition. This study provides a fermentation process condition to obtain the highest melanin production by *mel1*, which will allow it to be scaled in further studies.

## Results

### Effect of L-tyrosine, glucose, glutamic acid, l-DOPA, and copper sulfate on melanin production

According to the results obtained in the central composite design (CCD) (Table 1), the amount of melanin produced ranged from 14.26 up to 125.26 mg.g^−1^ ( milligram of melanin per gram of dry biomass), and the replicates at the center point resulted in short range of variation in melanin production, indicating that the response variation observed in the CCD experiment was due to different concentration of the factors in each experiment (Table 1).

**Table 1:** Central composite design ${2}^{5}$ matrix and experimental data for melanin production by *mel1* mutant

Experiment	Levels					Melanin (mg.g^−1^)
	X_1_	X_2_	X_3_	X_4_	X_5_	
1	1.4 (− 1)	0.8 (− 1)	3.5 (− 1)	1.4 (− 1)	7.5 (− 1)	56.832
2	3.4 (+ 1)	0.8 (− 1)	3.5 (− 1)	1.4 (− 1)	7.5 (− 1)	58.755
3	1.4 (− 1)	1.8 (+ 1)	3.5 (− 1)	1.4 (− 1)	7.5 (− 1)	38.771
4	3.4 (+ 1)	1.8 (+ 1)	3.5 (− 1)	1.4 (− 1)	7.5 (− 1)	32.218
5	1.4 (− 1)	0.8 (− 1)	7.5 (+ 1)	1.4 (− 1)	7.5 (− 1)	48.704
6	3.4 (+ 1)	0.8 (− 1)	7.5 (+ 1)	1.4 (− 1)	7.5 (− 1)	60.044
7	1.4 (− 1)	1.8 (+ 1)	7.5 (+ 1)	1.4 (− 1)	7.5 (− 1)	37.603
8	3.4 (+ 1)	1.8 (+ 1)	7.5 (+ 1)	1.4 (− 1)	7.5 (− 1)	28.043
9	1.4 (− 1)	0.8 (− 1)	3.5 (− 1)	3.4 (+ 1)	7.5 (− 1)	125.267
10	3.4 (+ 1)	0.8 (− 1)	3.5 (− 1)	3.4 (+ 1)	7.5 (− 1)	63.841
11	1.4 (− 1)	1..8 (+ 1)	3..5 (− 1)	3..4 (+ 1)	7..5 (− 1)	91..267
12	3..4 (+ 1)	1.8 (+ 1)	3.5 (-1)	3.4 (+ 1)	7.5 (− 1)	57.005
13	1.4 (− 1)	0.8 (− 1)	7.5 (+ 1)	3.4 (+ 1)	7.5 (− 1)	115.757
14	3.4 (+ 1)	0.8 (− 1)	7.5 (+ 1)	3.4 (+ 1)	7.5 (− 1)	91.309
15	1.4 (− 1)	1.8 (+ 1)	7.5 (+ 1)	3.4 (+ 1)	7.5 (− 1)	104.646
16	3.4 (+ 1)	1.8 (+ 1)	7.5 (+ 1)	3.4 (+ 1)	7.5 (− 1)	110.355
17	1.4 (− 1)	0.8 (− 1)	3.5 (− 1)	1.4 (− 1)	18 (+ 1)	45.948
18	3.4 (+ 1)	0.8 (− 1)	3.5 (− 1)	1.4 (− 1)	18 (+ 1)	89.603
19	1.4 (− 1)	1.8 (+ 1)	3.5 (− 1)	1.4 (− 1)	18 (+ 1)	37.776
20	3.4 (+ 1)	1.8 (+ 1)	3.5 (− 1)	1.4 (− 1)	18 (+ 1)	24.962
21	1.4 (− 1)	0.8 (− 1)	7.5 (+ 1)	1.4 (− 1)	18 (+ 1)	57.741
22	3.4 (+ 1)	0.8 (− 1)	7.5 (+ 1)	1.4 (− 1)	18 (+ 1)	53.941
23	1.4 (− 1)	1.8 (+ 1)	7.5 (+ 1)	1.4 (− 1)	18 (+ 1)	22.534
24	3.4 (+ 1)	1.8 (+ 1)	7.5 (+ 1)	1.4 (− 1)	18 (+ 1)	27.003
25	1.4 (− 1)	0.8 (− 1)	3.5 (− 1)	3.4 (+ 1)	18 (+ 1)	103.018
26	3.4 (+ 1)	0.8 (− 1)	3.5 (− 1)	3.4 (+ 1)	18 (+ 1)	98.555
27	1.4 (− 1)	1.8 (+ 1)	3.5 (− 1)	3.4 (+ 1)	18 (+ 1)	66.071
28	3.4 (+ 1)	1.8 (+ 1)	3.5 (− 1)	3.4 (+ 1)	18 (+ 1)	45.563
29	1.4 (− 1)	0.8 (− 1)	7.5 (+ 1)	3.4 (+ 1)	18 (+ 1)	70.81
30	3.4 (+ 1)	0.8 (− 1)	7.5 (+ 1)	3.4 (+ 1)	18 (+ 1)	61.692
31	1.4 (− 1)	1.8 (+ 1)	7.5 (+ 1)	3.4 (+ 1)	18 (+ 1)	33.84
32	3.4 (+ 1)	1.8 (+ 1)	7.5 (+ 1)	3.4 (+ 1)	18 (+ 1)	81.784
33	0.02 (− 2.38)	1.3 (0)	5.5 (0)	2.4 (0)	12.75 (0)	33.694
34	4.78 (+ 2.38)	1.3 (0)	5.5 (0)	2.4 (0)	12.75 (0)	47.708
35	2.4 (0)	0.11 (− 2.38)	5.5 (0)	2.4 (0)	12.75 (0)	78.86
36	2.4 (0)	2.49 (+ 2.38)	5.5 (0)	2.4 (0)	12.75 (0)	29.821
37	2.4 (0)	1.3 (0)	0.74 (− 2.38)	2.4 (0)	12.75 (0)	26.691
38	2.4 (0)	1.3 (0)	10.26 (+ 2.38)	2.4 (0)	12.75 (0)	51.922
39	2.4 (0)	1.3 (0)	5.5 (0)	0.02 (− 2.38)	12.75 (0)	14.266
40	2.4 (0)	1.3 (0)	5.5 (0)	6.85 (+ 2.38)	12.75 (0)	78.688
41	2.4 (0)	1.3 (0)	5.5 (0)	2.4 (0)	0.27 (− 2.38)	52.806
42	2.4 (0)	1.3 (0)	5.5 (0)	2.4 (0)	25.23 (+ 2.38)	43.573
43	2.4 (0)	1.3 (0)	5.5 (0)	2.4 (0)	12.75 (0)	70.588
44	2.4 (0)	1.3 (0)	5.5 (0)	2.4 (0)	12.75 (0)	84.072
45	2.4 (0)	1.3 (0)	5.5 (0)	2.4 (0)	12.75 (0)	66.512
46	2.4 (0)	1.3 (0)	5.5 (0)	2.4 (0)	12.75 (0)	79.893

**Fig. 1:**
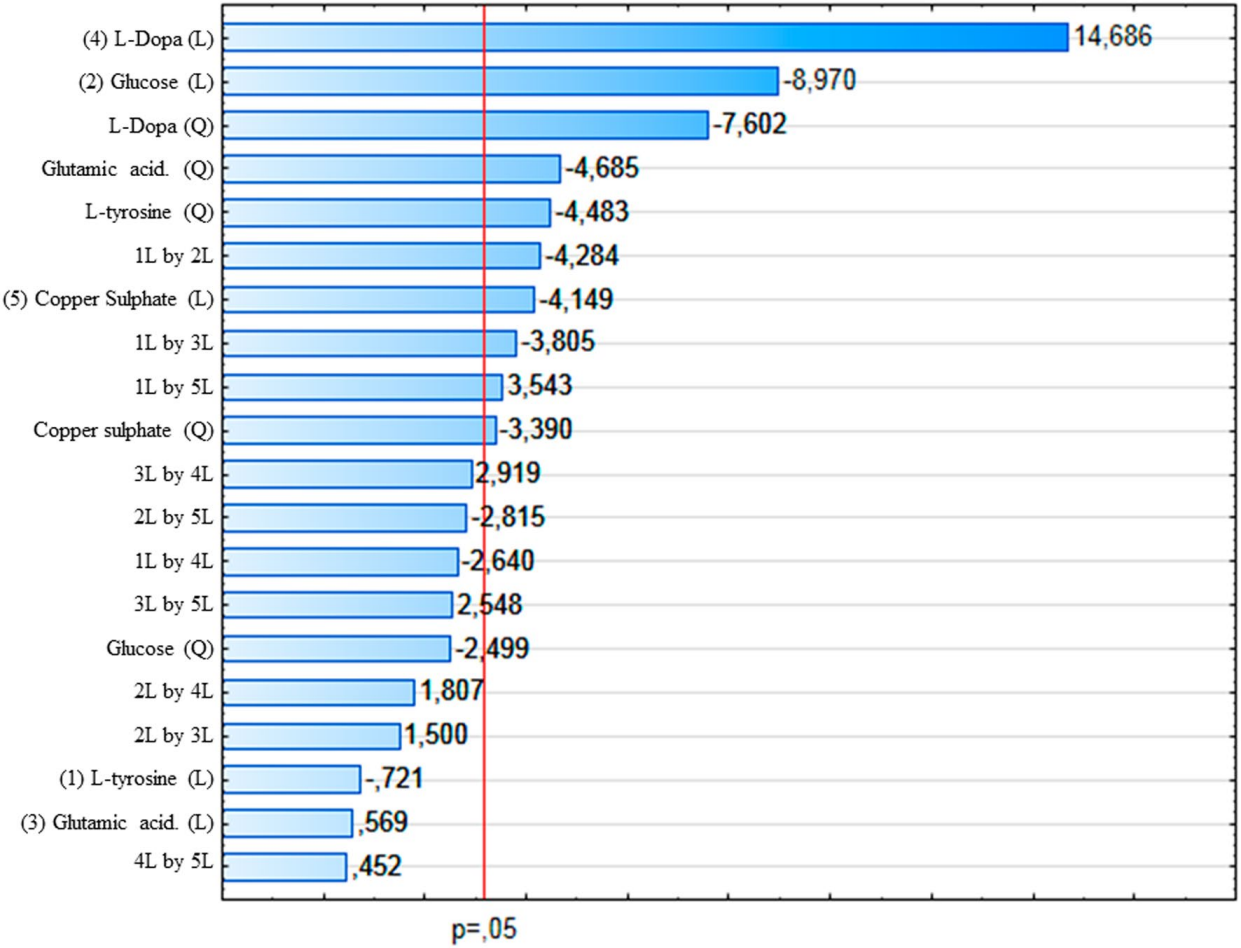
Pareto chart for the effects of each factor on melanin production by *mel1* mutant

The effects of the factors on the response and the significant levels (*p* < 0.05) can be explained by the Pareto chart (Fig. 1). It shows the estimated effects of each factor and the interactions between them. When considered in linear terms, l-DOPA, glucose, and copper sulfate showed the greatest influence on pigment production.

As can be observed in Fig. 1, l-DOPA had the most significant effect (*p* < 0.05) on melanin production by *mel1* mutant. Interestingly, l-DOPA was the only factor that exhibited a significant effect in the higher level on the response, indicating that in the presence of high concentrations of l-DOPA there was a greater melanin production. Glucose and copper sulfate also significantly influenced melanin production (*p* < 0.05); however, their effect was significant in the lower level, i.e., higher pigment production was obtained at lower concentrations of these compounds. It was also observed a significant interactive effect between copper sulfate and glucose (*p* < 0.05) where the lower-level value indicates that an increase in the concentration of any of these factors will decrease the pigment yield. When considered in linear terms, L-tyrosine and glutamic acid had no significant influence (*p* < 0.05) on the production of melanin.

Applying multiple regression analysis to the experimental data, the quadratic effects of the independent variables on the production of melanin were calculated and the following second-order polynomial equation was proposed to predict the optimal levels of the factors:

$$y= - 63.40+10.84{x}_{1}-5.45{x}_{1}^{2}-10.77{x}_{2}-12.14{x}_{2}^{2}+10.21{x}_{3}-1.43{x}_{3}^{2}+49.24{x}_{4}-3.99{x}_{4}^{2}+8.10{x}_{5}-0.5{x}_{5}^{2}+1.3{x}_{1}{x}_{2}+1.83{x}_{1}{x}_{3}-4.04{x}_{1}{x}_{4}+5.2{x}_{2}{x}_{4}-1.45{x}_{2}{x}_{5}+1.08{x}_{3}{x}_{4}-0.52{x}_{3}{x}_{5}-1.2{x}_{4}{x}_{5}$$

Where $y$ is the response of melanin production (mg. g ^−1^) and ${x}_{1}$, ${x}_{2}$, ${x}_{3}$, ${x}_{4}$ and ${x}_{5}$ are the coded values of the factors (L-tyrosine, glucose, glutamic acid, l-DOPA, and copper sulfate, respectively).

The statistical significance of the model was checked using analysis of variance (ANOVA). The ANOVA analysis of the second-order regression model demonstrated that the above-mentioned equation was highly significant. The calculated *F* value (4.93) was higher than the critical *F* value (2.01) and *p* < 0.05 (Table 2) and the lack-of-fit value for regression was not significant (*p* > 0.05), indicating that the model equation can be considered adequate to predict the melanin production by *mel1* within the range of the factors evaluated. Moreover, the coefficient of determination obtained by analysis (R^2^ = 0.7976) indicates that around 80% of the variability in the observed response values could be explained by the model, confirming a satisfactory adjustment of the proposed model to the experimental data.

**Table 2:** Analysis of variance (ANOVA) of the second-order model for the experimental data from the CCD design

Source of variation	Sum of squares	Degrees of freedom	Mean square	*F-*calc	*F-tab*	*p-value*
Regression	27,195.818	20	1359.79092	4.93	2.01	0.000127**
Residual	6899.4605	25	275.978418			
Lack of fit	6701.99	22	304.635714	4.63	8.66	0.11582
Pure terror	197.47	3	65.8249178			
Total	34,095.28	45				

R^2^ = 0.7976

^**^Significant *p* < 0.05

### Response surface analysis

**Fig. 2:**
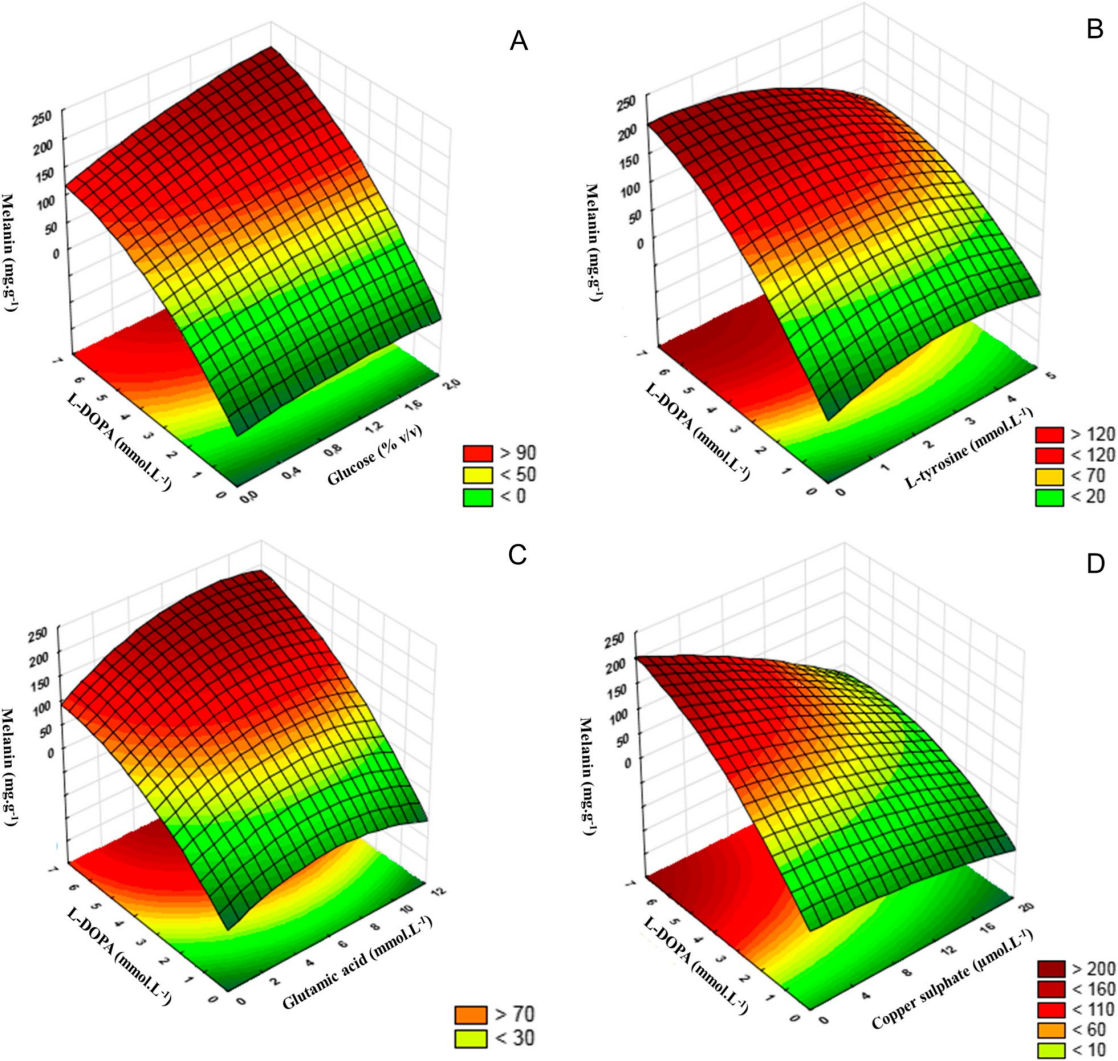
Response surface curves representing the interaction between the factors: **A** L-DOPA and glucose, **B** L-DOPA and L-tyrosine, **C** L-DOPA and glutamic acid, and **D** L-DOPA and copper sulfate on melanin production by *mel1* mutant

The response surface curves described by the regression model were drawn to illustrate the melanin production by *mel1* mutant in response to the interaction among tested factors, as well as to determine the optimum level of each factor for maximum pigment production (Fig. 2). In each plot, two factors varied within their experimental range, while the other three factors remained constant at the central point.

Analyzing the glucose and l-DOPA interaction on melanin production (Fig. 2A), it is possible to verify that melanin yields increased with increasing l-DOPA concentration and greater production was achieved at the highest concentration of l-DOPA and glucose (7 mmol. L^−1^ and 2 mmol.L^−1^, respectively).

Concerning the interaction between l-DOPA and L-tyrosine, the Fig. 2B shows that greater production of melanin was also obtained at the higher concentration of l-DOPA (7 mmol. L^−1^), but with the l-tyrosine concentration at its lowest level (< 1 mmol.L^−1^). Increasing l-tyrosine concentration decreased melanin yield even in the high concentration of l-DOPA. Figure 2C reveals that the interaction of l-DOPA with glutamic acid resulted in maximum melanin production when l-DOPA and glutamic acid were set at their highest level (7 mmol. L^−1^ and 12 mmol.L^−1^, respectively). The interaction between l-DOPA and copper sulfate, shown in Fig. 2D, confirms that melanin production increases in the high concentration of -DOPA (7 mmol. L^−1^), whereas copper sulfate must be at its lowest concentration. From these analyses, the following optimal values of the factors tested were obtained through desirability function: 0.02 mmol.L^−1^ l-tyrosine, 1.8% (v/v) glucose, 10.26 mmol.L^−1^ glutamic acid, 6.85 mmol.L^−1^ l-DOPA and 0.27 µmol.L^−1^ copper sulfate.

### Experimental validation of the model

**Fig. 3:**
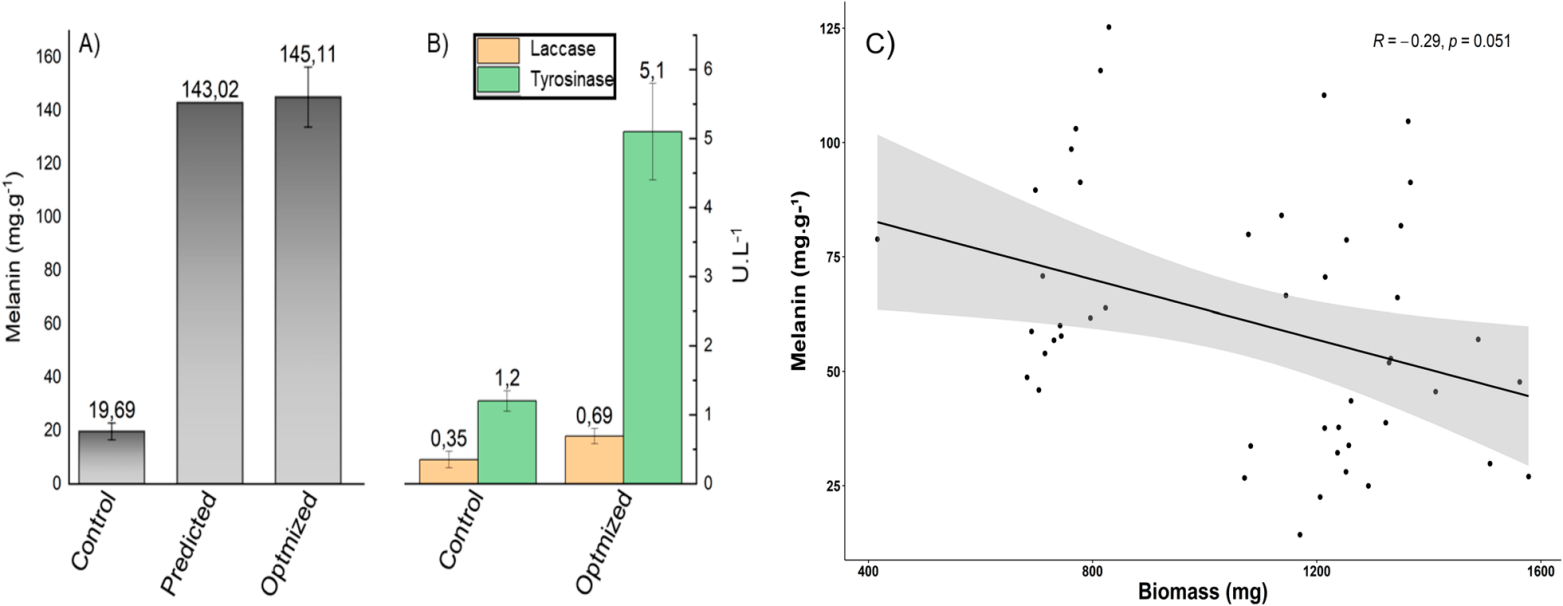
Experimental validation of the model. **A** Melanin production by *mel1* mutant after grown under optimized and non-optimized conditions compared to that predicted by the model. **B** Tyrosinase e laccase activities after growth of the *mel1* mutant under

To experimentally validate the model, the *mel1* mutant was grown in a minimal culture medium supplemented with each factor at its optimal concentration, and melanin production of 145 mg.g^−1^ was obtained, representing an increase of 640% compared to the non-optimized condition (Fig. 3A). The melanin yield predicted by the model (143.02 mg.g^−1^) differs from the experimental one by only 1.5% (Fig. 3A), demonstrating a high degree of accuracy of the model, which indicates the reliability and validity of the proposed model. This result also indicates that the parameters optimized by the RSM are reliable and that the model is adequate for estimating the experimental value of the response in future observations.

To assess the relationship between melanin production and the synthesis pathway of this pigment in the *mel1* mutant, the activity of tyrosinase and laccase was evaluated under optimized and non-optimized conditions (Fig. 3B). The enzyme activities in optimized conditions were higher compared to control. Laccase activity enhanced from 0.35 to 0.69 U.L^−1^ whereas the tyrosinase activity showed a greater increase from 1.2 to 5.1 U.L^−1^. These results may indicate that tyrosinase is the main enzyme involved in melanin synthesis by *mel1* mutant. Also, the correlation between melanin production and biomass yield was analyzed using Spearman's rank-order correlation (Fig. 3C). It is possible to verify that melanin yield is weakly correlated to biomass production ($R=-0.29, p>0.05$). This result reveals that the melanin production could not be explained as a response to higher biomass production, confirming the role the key factors optimized by the CCD experiment had on melanin production.

## Discussion

This study showed that all factors influenced the production of melanin by *mel1* mutant (Table 1). We observed that l-DOPA, glucose, and copper sulfate exerted a significant effect on yield whereas l-DOPA was the only factor that resulted in a significant effect on a higher level of pigment production (Fig. 1). This result may be due to l-DOPA acting as a substrate for melanin synthesis [20] and also confirms that the *mel1* mutant produces DOPA-melanin, as we previously reported [16]. Similarly, Eisenman et al. [19] also demonstrated the positive effect of l-DOPA for melanization of *Cryptococcus neoformans*, which requires exogenous addition of L-DOPA for melanin synthesis to take place. A similar study showed that *Sporothrix schenckii* strains are incapable to synthesize melanin without l-DOPA supply in the culture medium [24].

Surprisingly, l-tyrosine did not show a significant effect on melanin production by *mel1*, even though this amino acid is very well documented as a precursor for melanin synthesis [25, 26]. As reported by Zou et al. [26] and Sun et al. [21], l-tyrosine plays an important role in the production of melanin by *Auricularia auricula*, which exhibited higher pigment production when the culture medium was supplemented with increasing concentrations of l-tyrosine. In contrast, the preference for l-DOPA over l-tyrosine for melanin production may be explained by the type of melanin produced by the *mel1* mutant [16], and also because one more enzymatic step is needed to form DOPA-melanin when tyrosine is used as a substrate compared to the synthesis of this pigment using DOPA as a precursor [27, 28]. First, tyrosine is enzymatically converted to DOPA, then to dopaquinone to ultimately form melanin [20]. On the other hand, supplementation of the medium with l-DOPA may represent a cell strategy to save energy in this biosynthesis pathway.

Our results also revealed a fourfold increase in tyrosinase activity in the optimized culture medium compared to the control (Fig. 3B). The explanation for this result may be related to the four oxidation states of the active site of the tyrosinase enzyme: met-tyrosinase, deoxy-tyrosinase, oxy-tyrosinase, and deact-tyrosinase [29]. The native state of this enzyme is met-tyrosinase, in which it can interact with monophenols, but no catalytic reaction takes place. The met-tyrosinase is a non-active state of the enzyme since it is necessary for the presence of a diphenol that binds to the active site of the met-tyrosinase and then the enzyme is reduced to the deoxy-tyrosinase state. In this state, the enzyme can bind to molecular oxygen to finally reach its oxy-tyrosinase state, which represents the active state of the enzyme. Oxy-tyrosinase can oxidize phenols to their corresponding o-quinones by both monophenol oxidase and diphenol oxidase mechanisms [29]. Thus, the L-DOPA (diphenol) present in the optimized condition was used for tyrosinase enzyme activation, which resulted in increased melanin production in response to higher tyrosinase activity. This hypothesis explains the significant positive effect of l-DOPA on pigment production due to its use as a preferential substrate for melanin synthesis by the *mel1* mutant.

We noticed an interaction between l-DOPA and glucose since the greatest production of melanin obtained at a high concentration of l-DOPA only occurred when glucose was also set in high concentrations (Fig. 2A). According to the literature, the carbon source and its concentration in the culture medium can directly affect the biosynthesis of secondary metabolites in fungi [30], including melanin pigment. For example, Sun et al. [21], reported that glucose concentration affects both mycelial growth and melanization in *A. auricula*, as this carbon source provides energy for cellular activities as well as melanin biosynthesis in this fungus. This explanation can be applied to our results, as the increase in pigment production at high DOPA concentration requires a large amount of energy, which is provided by the high concentration of glucose. Although glutamic acid did not show a positive effect on pigment production (Fig. 1), it is also required in high concentrations to obtain greater melanin production in the presence of l-DOPA (Fig. 2C). A similar result was reported by Wu et al. [31], which observed an increased production of extracellular melanin by *Rhizobium radiobacter* with an increasing concentration of glutamic acid. These results can be explained by the stimulation of tyrosinase enzyme activity, which consequently increases melanin synthesis, as reported by Palumbo et al. [32]*.*

Conversely, lower concentrations of copper sulfate were necessary to obtain greater melanin production in the presence of l-DOPA (Fig. 2D). Although copper sulfate is widely known as an inducer of tyrosinase and laccase, enzymes responsible for melanization in fungi [20], studies have shown that the increased production of melanin due to the presence of copper depends on the concentration of metal in the culture medium. Galhaup and Haltrich [24] demonstrated that increasing the concentration of copper in the culture medium up to a certain value resulted in a considerable increase in laccase activity as well as melanin content in *Trametes pubescens*. In this work, it was also observed, that higher concentrations of copper did not promote greater melanization of the *mel1* mutant. Finally, the weak correlation between biomass production and melanin yield (Fig. 3C) confirms that the result of melanin yield is due to the optimization success of the key factors in the culture medium instead of a result of a higher amount of melanized biomass.

## Conclusion

In this study, the response surface methodology showed to be effective and reliable in selecting the significant factors and determining their optimal concentration to obtain greater melanin production by the *mel1* mutant. Among the factors analyzed, l-DOPA, glucose, and copper sulfate significantly affected melanin production whereas l-DOPA was the only factor that exerted a positive effect on pigment yield.

After optimization, the following medium composition was established: glucose (1.8%, v/v, l-DOPA (6.85 mmol.L^−1^),: L-tyrosine (0.02 mmol.L^−1^), glutamic acid (10.26 mmol.L^−1^), and copper sulfate (0.27 µmol.L^−1^). In this condition, the melanin synthesis was increased from 19.69 to 145.11 mg.g^−1^, resulting in a 640% increase compared to the condition before the optimization. Besides, we also showed that tyrosinase activity was higher in the optimized medium, probably because l-DOPA is the substrate required for enzyme activation, which would explain the increased production of pigment in this condition. In addition, this study clearly showed a strong response to tyrosinase enzyme activity, which enhanced its activity 4 times under the optimized condition compared to control. Thereby, the optimization of the culture medium composition will make it possible to scale the process to obtain large amounts of pigment for a future biotechnological application of the melanin produced by the *mel1* mutant.

## Materials and methods

### Strain and growth conditions

The present study was carried out with the highly melanized mutant from *A. nidulans,* denominated *mel1*, which also has auxotrophy for inositol [16]. This mutant was isolated by Pombeiro (1991) after NQO (4-nitroquinoline-1-oxide) mutagenesis in a strain of *A. nidulans* [38]. This strain exhibits colonial growth with the production of dark pigment (visible on the back of the colony) and sparse conidiation with green-colored spores. In addition, genetic analysis of the *mel1* mutant showed that only one gene, called melC, is involved in melanin production and was located on chromosome (linkage group) VII [38]. This fungus belongs to the culture collection of the Filamentous Fungi Laboratory at the Department of Biochemistry and Organic Chemistry, the Institute of Chemistry, São Paulo State University – UNESP, Araraquara -Brazil.

The *mel1* mutant was first grown in a solid minimal medium, as described by Cove [33], supplemented with glucose (55 mmol. L^−1^), sodium nitrate (70 mmol. L^−1^), steep liquor (1% v/v), inositol (20 mg. L^−1^) for 5 days at 37ºC to obtain a conidial suspension.

Cultivation of the *mel1* mutant for pigment production was carried out in two steps, as described by Sponchiado et al. [23]. In the first step, the *mel1* mutant was grown in 500 mL Erlenmeyer flasks containing 200 mL of minimal culture medium [33] supplemented with 55 mmol. L^−1^ glucose, 70 mmol. L^−1^ sodium nitrate, 1% (v/v) steep corn liquor, 20 mg. L^−1^ inositol inoculated with 10^6^ conidia. mL^−1^ and incubated at 29 °C in a rotary shaker at 225 rpm for 48 h. After incubation, the mycelium was collected by centrifugation at 2576 g for 10 min and it was used as inoculum in the 2nd step. In this step, 20 mL of mycelial mass was transferred to Erlenmeyer flasks (500 mL) containing 200 mL of liquid minimal medium [33] supplemented with 70 mmol. L^−1^ sodium nitrate, 1% (v/v) steep corn liquor, 20 mg. L^−1^ inositol and tween 80 (0,2%, v/v). In this medium, l-tyrosine, glucose, glutamic acid, l-DOPA, and copper sulfate were added in their respective concentrations, according to the experimental design. After growth for 72 h at 29 °C in a rotary shaker at 225 rpm, the mycelium was collected by vacuum filtration and the pigment present in the mycelium was then extracted.

### Optimization of melanin production through Response Surface Methodology – RSM

A 2^5^ central composite design was applied to identify the effect of l-tyrosine, glucose, glutamic acid, l-DOPA, and copper sulfate (independent variables) on the melanin production (dependent variable) by *mel1* mutant. This strategy allows us to assess both the effect of the factors and the axial points α = (2^5^)^1/4^ on melanin production. The five factors, the axial points to each factor, and 4 replications at the center points lead to a total of 46 experiments, where each one of the levels of the factors was established according to Table 3. The concentrations of factors used in this design were chosen based on previous studies and the axial points to each factor were calculated as α = (2^5^)^1/4^.

**Table 3:** The levels of five independent variables employed in CCD. Coded factors are described in parentheses

		**Level**				
**Factor**	**Symbol**	-α*	**Lower**	**Center Point**	**Higher**	+ α*
L-tyrosine (mmol.L^−1^)	X_1_	0.02 (− 2.38)	1.4 (− 1)	2.4 (0)	3.4 (+ 1)	4.78 (+ 2.38)
Glucose (% v/v)	X_2_	0.11 (− 2.38)	0.8 (− 1)	1.3 (0)	1.8 (+ 1)	2.49 (+ 2.38)
Glutamic acid (mmol.L^−1^)	X_3_	0.74 (− 2.38)	3.5 (− 1)	5.5 (0)	7.5 (+ 1)	10.26 (+ 2.38)
L-DOPA (mmol.L^−1^)	X_4_	0.02 (− 2.38)	1.4 (− 1)	2.4 (0)	3.4 (+ 1)	6.85 (+ 2.38)
Copper sulfate (µmol.L^−1^)	X_5_	0.27 (− 2.38)	7.5 (− 1)	12.75 (0)	18 (+ 1)	25.23 (+ 2.38)

^*^α = axial point

A set of 46 experiments, including 4 replicates at the center point, were performed, according to the matrix shown in Table 1. The experiments were performed to obtain a second-order model to predict the amount of melanin production in response to different factors (independent variables). The quadratic model for predicting the optimal point was expressed as follows:

$$y= {\beta }_{0} + \sum_{i=1}^{k}{\beta }_{i}{x}_{i} + \sum \sum_{i<j}^{k}{\beta }_{ij}{x}_{i}{x}_{j} + \sum_{i=1}^{k}{\beta }_{ii}{x}_{i}^{2}$$

Where $y$ represents the predicted response, ${\beta }_{0}$ is the intercept, ${\beta }_{i}$ ${\beta }_{ij}$, and ${\beta }_{ii}$ are the measures of the effects of variables ${x}_{i}$, ${x}_{i}{x}_{j}$, and ${x}_{i}^{2}$, respectively.

The response surface curves were constructed from the quadratic model to assess the effects of variables individually and in combination and to determine their optimal levels of each factor for maximum pigment production.

### Melanin extraction and quantification

The melanin from mycelium was extracted according to the procedure described by Sava et al. [34], with modifications made by Gonçalves et al. [16]. Firstly, the mycelium was ground to powder using liquid nitrogen, and the pigment was extracted with 1 mol NaOH, at a ratio of 1:30 (w/v), after autoclaved at 121 °C, 1 kgf/cm^2^ for 10 min. The supernatant containing melanin solubilized was collected by centrifugation at 2576 g for 10 min and the precipitate was re-dissolved with 1 mol NaOH (1:30 w/v). All the previous steps were repeated until complete melanin extraction. The solubilized melanin was acidified to pH 2.5 with concentrated HCL kept for 72 h at room temperature and centrifuged at 2576 g for 15 min. The precipitate obtained was washed with distilled water and dried at 60 °C for 24 h. For quantification of melanin, the dry pigment was re-dissolved in 0.5 mol NaOH (1 mg.mL^−1^) and the concentration of melanin was determined spectrophotometrically at 540 nm from a standard curve synthetic DOPA-melanin (Sigma-Aldrich).

### Enzymatic activity assay

Tyrosinase and laccase enzymes assays were performed with the crude extract obtained from mycelia grown under optimized and non-optimized conditions. The mycelium was ground with liquid nitrogen and suspended in extraction buffer (Tris-HCL 50 mmol.L^−1^, EDTA 1 mmol.L^−1^, PMSF mmol.L^−1^) in the proportion of 1:2 (m/v), followed by centrifugation at 9000 *xg* for 10 min [35]. The resulting supernatant considered a cell-free extract was used immediately to determine enzymatic activity. All the assays were done in triplicates.

Tyrosinase activity was determined by monitoring the conversion of l-DOPA to dopachrome by the absorbance increase at 475 nm. Reaction mixture containing 0.6 mL cell-free extract, 1 mL sodium phosphate buffer (0.1 mol.L^−1^) pH 6.8, and 0.4 mL of L-DOPA solution (20 µmol.L^−1^ of l-DOPA prepared in 0.1 mol.L^−1^ sodium phosphate buffer pH 6.8) was incubated at 30 °C for five minutes. The cell-free extract heated at 100 °C for 5 min before adding to the reaction mixture was used as control. One unit of tyrosinase activity was defined as the amount of enzyme that catalyzes the formation of 1 µmol of dopachrome per minute at pH 6.8 at 30 °C under the reaction condition, using the molar absorption coefficient of dopachrome at 475 nm (${3600 M}^{-1}{cm}^{-1}$) [36]. The assays were carried out in triplicate and the results were expressed as enzyme units per liter (U.L^−1^).

Laccase activity was spectrophotometrically determined by the oxidation of Syringaldazine to its quinone form at 525 nm, as described by Szklarz et al. [37] with modifications. The enzymatic assay was prepared with 0.6 mL cell-free extract, 1 mL phosphate-citrate buffer 0.05 mol.L^−1^ pH 5.0, and 0.4 mL of Syringaldazine solution (0,1% of Syringaldazine prepared in ethanol) and incubated for 10 min at room temperature. Heating the cell-free extract at 100 °C for 5 min before adding it to the reaction mixture was used as a control. One unit of laccase activity was defined as the amount of enzyme required to oxidize 1 mmol of Syringaldazine per minute under the reaction condition, using the molar absorption coefficient for the product ($6.5 x {10}^{4} {M}^{-1}{cm}^{-1}$). The assays were carried out in triplicate and the results were expressed as enzyme units per liter (U.L^−1^).

## Statistical analysis

The results from the CCD experiment were interpreted using the Statistica v.10 Software and all the other results were analyzed using the R Statistical Environment Platform. The analysis of variance (ANOVA) was used to determine the statistical significance of each factor on the response variable ($$p<$$ 0.05). The model fit was expressed by the coefficient of determination ($${R}^{2}$$). The quadratic model of the variables was depicted as surface response curves and the experimental validation of the model was performed using the desirability of variables for maximum response. Shapiro-Wilk test was used to check the normality distribution of biomass production and melanin yield data ($$p<0.05)$$, and then Spearman’s rank correlation coefficient was used to determine the strength and direction of their relationship.

## Supplementary Information

**Additional file 1.** Experimental dataset obtained from Central Composite Design for melanin production by mel1 mutant and the activity of tyrosinase and laccase enzymes.

## Data Availability

All data generated and analysed during this study are included in this published article.
